# Extracellular Vesicles as a Translational Approach for the Treatment of COVID-19 Disease: An Updated Overview

**DOI:** 10.3390/v15101976

**Published:** 2023-09-22

**Authors:** Enrica Serretiello, Andrea Ballini, Annafrancesca Smimmo, Marina Acunzo, Mariarosaria Raimo, Stefania Cantore, Marina Di Domenico

**Affiliations:** 1 Department of Precision Medicine, University of Campania “Luigi Vanvitelli”, 80138 Naples, Italy; enrica.serretiello@unicampania.it (E.S.); annafrancescasmimmo@gmail.com (A.S.); marina.acunzo@hotmail.it (M.A.); raimomariarosaria83@outlook.it (M.R.); stefaniacantore@pec.omceo.bari.it (S.C.); marina.didomenico@unicampania.it (M.D.D.); 2 Department of Clinical and Experimental Medicine, University of Foggia, 71122 Foggia, Italy

**Keywords:** COVID-19, extracellular vesicles, exosomes, mesenchymal stromal cell, mesenchymal stromal cell-derived extracellular vesicles, exosome-based vaccines, precision medicine

## Abstract

Severe acute respiratory syndrome coronavirus 2 (SARS-CoV-2) caused a global pandemic in the years 2020–2022. With a high prevalence, an easy route of transmission, and a long incubation time, SARS-CoV-2 spread quickly and affected public health and socioeconomic conditions. Several points need to be elucidated about its mechanisms of infection, in particular, its capability to evade the immune system and escape from neutralizing antibodies. Extracellular vesicles (EVs) are phospholipid bilayer-delimited particles that are involved in cell-to-cell communication; they contain biological information such as miRNAs, proteins, nucleic acids, and viral components. Abundantly released from biological fluids, their dimensions are highly variable, which are used to divide them into exosomes (40 to 150 nm), microvesicles (40 to 10,000 nm), and apoptotic bodies (100–5000 nm). EVs are involved in many physiological and pathological processes. In this article, we report the latest evidence about EVs’ roles in viral infections, focusing on the dual role of exosomes in promoting and inhibiting SARS-CoV-2 infection. The involvement of mesenchymal stromal/stem cells (MSCs) and MSC-derived EVs in COVID-19 treatment, such as the use of translational exosomes as a diagnostical/therapeutic approach, is also investigated. These elucidations could be useful to better direct the discovery of future diagnostical tools and new exosome-derived COVID-19 biomarkers, which can help achieve optimal therapeutic interventions and implement future vaccine strategies.

## 1. Introduction

### 1.1. SARS-CoV-2 Structure

The infectious disease caused by severe acute respiratory syndrome coronavirus type 2 (SARS-CoV-2) was reported in December 2019, in Wuhan, China [1]. On 11 February 2020, the World Health Organization (WHO) defined it as Coronavirus Infectious Disease 2019 (COVID-19) and declared its pandemic status on 11 March 2020 [2]. As of 21 June 2023, the WHO reported 768,187,096 confirmed SARS-CoV-2 cases from the pandemic, and 6,945,714 deaths worldwide [2]. The burden of the pandemic had globally affected psychological and socioeconomic conditions, but above all, the sustainability of public health systems [3]. SARS-CoV-2 is part of the beta groups of coronaviruses (HCoV-HKU1, HCoV-OC43, SARS-CoV, and MERS-CoV). In particular, it presents 70% homology with SARS-CoV [4]. The first cases of severe acute respiratory syndrome coronavirus (SARS-CoV) were identified in south central China in 2003, with a mortality rate of 10%. Ten years later, cases of Middle East respiratory syndrome coronavirus (MERS-CoV) were identified in Saudi Arabia, with a mortality rate of 37% [5,6]. 

Some coronavirus members are zoonotic viruses that are particularly capable of performing spillovers. SARS-CoV-2 presents a diameter range of 80–120 nm with a spherical shape, an envelope, and a positive single-stranded RNA [7,8]. The envelope is coated with spike-like glycoproteins, which appear as the characteristic crown form when being viewed under an electron microscope. The large genome, of about 30 kilobases, codes for the structural membrane (M), the envelope (E), the spike protein (S), and several non-structural proteins (nsps). M, E, and S are fundamental for several viral functions, such as host infection [9], membrane fusion [10], viral assembly [11], morphogenesis, and release of virus particles [12]. On the other hand, nsps are involved in viral replication and transcription functions [13,14]. 

The transmembrane S proteins protrude from the viral envelope and consist of three segments: an intracellular tail, a single-pass transmembrane, and a large ectodomain. The large ectodomain consists of a receptor-binding domain (RBD) in the S1 subunit and the membrane-fusion subunit (S2). The RBD domain is responsible for the recognition of the host cell receptor, which is specific for each coronavirus, and angiotensin-converting enzyme 2 (ACE2) has been identified as being fundamental for the first step of viral infection [15,16]. The S proteins and their interactions with the related receptors represent a key for eliciting antibodies and a principal target for new therapeutic approaches.

### 1.2. COVID-19 Features

**Figure 1:**
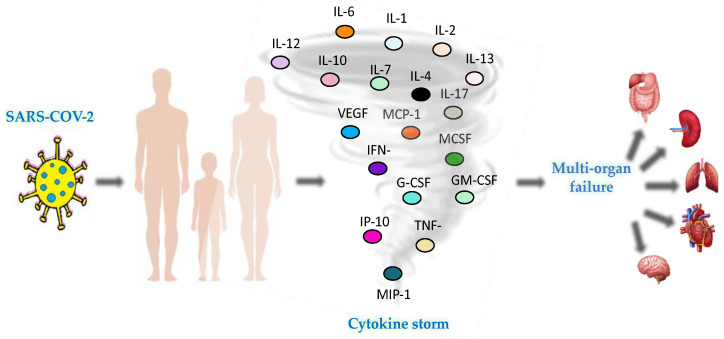
SARS-CoV-2 infection implications. COVID-19 pathogenesis can evolve into a “cytokine storm”, resulting in the release of inflammatory molecules and causing multi-organ failure [10,11,12].

Clinical manifestations of COVID-19 are extremely different, varying from asymptomatic or mild sickness with fever, cough, sore throat, loss of smell and taste, tiredness, and gastrointestinal disturbances to severe respiratory failure and even death. Respiratory failure may require mechanical ventilation support, risking the evolution into acute respiratory distress syndrome (ARDS). SARS-CoV-2 is able to infect different host cells, trigger a hyper-activation of the immune system with consistent inflammatory hyper-response and subsequent cytokine storm activation, along with widespread organ inflammation, leading to endothelialitis and affecting the vascular system. Therefore, COVID-19 patients are subjected to alterations in the coagulation process, such as disseminated intravascular coagulation (DIC), with possible consequences of stroke, heart attack, and organ damage, particularly in the pulmonary tissue (Figure 1). 

Thus, multiple organ dysfunction (MOD) that is caused by viral dissemination in endothelial cells can occur [17,18,19,20]. Moreover, predisposition to secondary bacterial, viral, and fungal infections has been reported [21,22]. The presence of comorbidities or an immunocompromised system exposes patients to severe COVID-19-related complications. In fact, advanced disease stages exhibit peculiar characteristics reported for idiopathic pulmonary fibrosis (IPF), chronic obstructive pulmonary disease (COPD), and lung cancer [23].

### 1.3. Extracellular Vesicles (EVs)

In 1967, EVs were discovered and identified as ‘‘platelet-dust” [24]. Successfully, the terms ‘‘extracellular vesicle” [25] and ‘‘exosome” [26] were used for the first time in 1971 and 1981, respectively. In 1983, EVs were biologically identified as transferrin carriers [27,28,29].

In 1987, R. M. Johnstone published an article entitled “Vesicle formation during reticulocyte maturation. Association of plasma membrane activities with released vesicles (exosomes)” [30]. In 2011, the term EVs was used as a collective term to describe cell-derived particles enclosed by a lipid bilayer, but only in the last 10 years that scientific research had proceeded to obtaining insight about their characterization with the introduction of new performing and cutting-edge technologies [29,30,31]. EVs are micro-particles of cellular origin that contain essential genetic and structural cellular components, such as mRNA and microRNA, organelles, receptors, and lipids. As they release their content to target cells, they turn out to be fundamental for intercellular cross-talk. Inter-cell communication is a fundamental mechanism to establish correct cell growth and acts as a defense strategy against pathogens [32]. Therefore, it is not surprising that EVs are involved in both physiological and pathological processes. In a pleiotropic manner, they affect fundamental biological processes, such as stem cell maintenance and plasticity [33], blood coagulation [34], immune surveillance by activating the immune responses or suppressing the inflammatory process, and injured tissue repair [35] by activating cell surface receptors and/or lipid ligands and transferring their contents (effector/regulatory molecules) to recipient cells (Figure 1) [36]. On the other hand, EV involvement is also reported to be involved in pathological conditions, such as cancer development, metastasis, and anti-tumor or pro-tumor immunity.

EVs can be produced by cells from all three domains of Bacteria, Archaea, and Eukarya, probably to preserve their evolutionary function of cell-to-cell communication mediators. In fact, EVs are isolated from several biological matrixes: plasma, liquor, saliva, urine, and milk [33,34,35,36,37].

#### EVs Nomenclature

**Figure 2:**
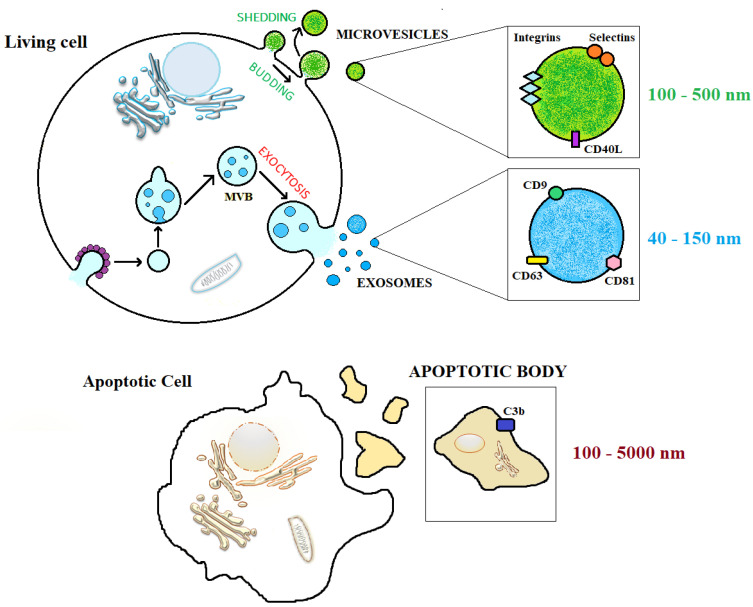
Extracellular vesicle structure and biogenesis.

Based on their size and the formation process, EVs are commonly defined and classified as exosomes, microvesicles (MVs), and apoptotic bodies (ABs) [38]. Exosomes are a heterogeneous group of vesicles with dimensions ranging from 40 to 150 nm. Their formation process can be dependent or independent of endosomal sorting complex required for transport (ESCRT) and involves the invagination of the plasma membrane to form endosomes, which is followed by the invagination of the endosomal membrane, forming a multivesicular body (MVB) with intraluminal vesicles inside. The fusion of MVBs with the cell membrane allows exosomes to be released outside [39,40]. MVs are larger than exosomes with sizes ranging from 40 to 10,000 nm. MVs are instead generated by plasmatic membrane budding, which requires the proteic and lipidic rearrangement of the membrane, which increases the phosphatidylserine and calcium concentration [32]. Apoptotic bodies are released by cells undergoing programmed cell death and have sizes of 100–5000 nm. The biogenesis mechanisms of EVs are presented in Figure 2.

Exosome synthesis occurs due to the inward budding of the endosomal membrane in three steps: (i) plasmatic membrane invagination to form endosomes; (ii) invagination of the endosomal membrane to form multivesicular bodies (MVBs) containing intraluminal vesicles (exosomes); and (iii) MVBs can follow two fates, either fusing with lysosomes for degradation of their contents or fusing with the cellular membrane to release their contents into the extracellular environment. Microvesicles (shedding vesicles) are synthesized by the outward budding of the plasma membrane. Apoptotic bodies are formed as result of membrane extroflexion of an apoptotic cell [29,30]. The exact and unambiguous nomenclature of EVs is a controversial and constantly evolving issue. It is necessary to mention the ISEV (International Society for Extracellular Vesicles) considerations expressed in the “Minimal information for studies of extracellular vesicles” (MISEV) 2018 paper [28]. ISEV defined “extracellular vesicle” (EV) as particles naturally released by cells, delimited by a lipid bilayer and unable to replicate. Recent studies highlight how the world of EVs has been further expanded following their recent characterizations. Indeed, small and large exosomes termed Exo-S (40–80 nm) and Exo-L (80–150 nm) were reported. On the other hand, small microvesicles called ARMM (40–100 nm) were recently discovered and reported distinguished from classical large-microvesicles (~150–1000 nm) and large oncosomes (1–10 µm). Other than the canonical EVs classification, autophagic EVs, stressed EVs, and matrix vesicles were added to this big nanoparticle family. Caused by a lack of consensus about the specific markers of EV subtypes, to discriminate by endo-some-origin “exosomes” and plasma membrane-derived “ectosomes” (microparticles/microvesicles), EVs should be identified by the biogenesis origin, which is difficult to identify. It is urgent to consider the use of operational terms for EV subtypes that refer to (a) the physical characteristics of EVs (size or density) and (b) the biochemical composition and descriptions of conditions or cell of origin, due to the pollution of the terms previously coined such as exosome and microvesicle [40,41,42,43].

Chaperones and protein kinases are involved in exosome biogenesis and secretion. The chaperone Cdc37 assists protein kinase folding and is crucial in exosome secretion, thus regulating autophagy and mitophagy. Other kinase members such as autophagy-activating kinase ULK1, lipid kinase VPS34, and membrane-bound kinase SRC are fundamental for exosome formation and release. Protein S-palmitoylation is crucial for sorting cargo proteins into EVs, thus contributing to their delivery to the recipient cell.

The Autophagosome, the vacuole obtained from the sequestration of a portion of the cytoplasm during autophagy, can fuse with endosomes (MVEs). The so generated “amphisomes”, fusing with the plasma membrane secretes autophagic EV cells that contain endosome- and autophagosome-derived molecules (LC-3 and CD63) [44,45,46,47].

Therefore, EVs differ from each other in terms of size, biogenesis process, biological characterization, and surface biomarkers and cargos, as reported in Table 1. Their high variability in features makes EVs peculiar and fundamental to cell–cell communication.

**Figure 3:**
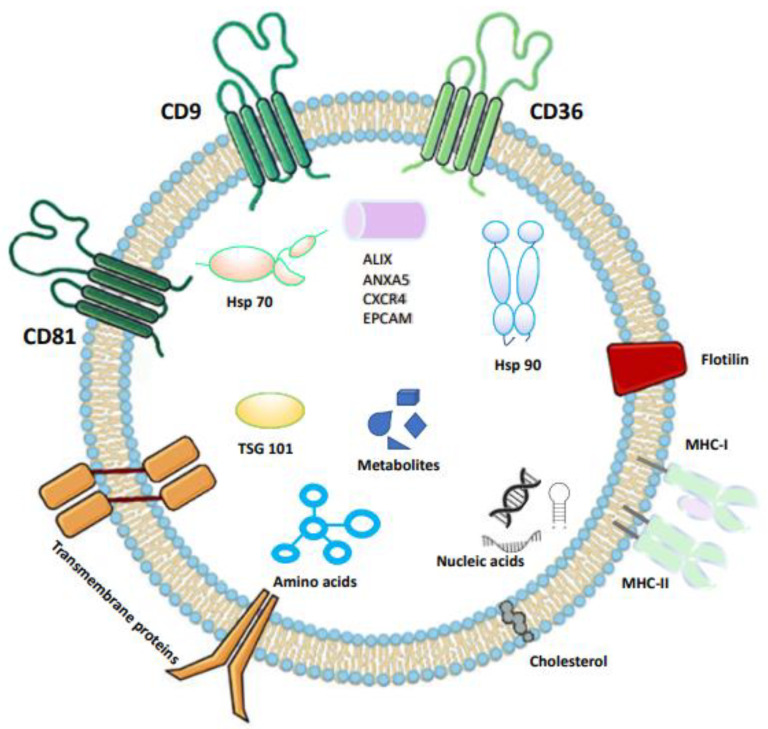
Exosome biomarkers and cargos. In general, the reported exosome biomarkers are involved in several biological effects, such as cell survival, proliferation, and migration; metabolic pathways; receptor–ligand signaling; and immune cell decoy and immuno-suppression. Proteins such as annexin, flotillin, Ras-associated binding (Rab) protein, and nucleotide guanosine triphosphate hydrolase (GTPase) are involved in transport and fusion membrane mechanisms. Alix, Tsg101, and HSP, which are components of the ESCRT complex, are involved in multivesicular biogenesis. Integrin and tetraspanine, such as CD9, CD53, CD63, CD81, and CD82, are involved in cellular mechanisms and cancer promotion. In particular, CD9 is involved in gene expression regulation, cellular metabolic reprogramming, and recipient cell apoptosis. CD81 is involved in cancer progression, including metastatic spread, organotropism, polymorphonucleate (PMN) formation, and angiogenesis. Abbreviations: motility-related protein-1 (CD9); human leucocyte surface antigen (CD53); cluster of differentiation 36 (CD36); cluster of differentiation 81 (CD81); cluster of differentiation 82 (CD82); major histocompatibility complexes I and II (MCH1-MCH2); heat shock protein 70 (Hsp70); heat shock protein 90 (Hsp90); tumor susceptibility gene 101 (TSG 101); ALG-2-interacting protein X (ALIX); annexin (ANXAS); C-X-C chemokine receptor type 4 (CXCR4); and epithelial cell adhesion molecule (EPCAM) [38,39,40,41,42,43,44,45,46,47,48].

The content of EVs depends on the cellular context, the tissue of origin, and the immune set-point. Their versatility and highly informative content make them promising biomarkers. EVs are able to improve cell–cell communication through juxtracrine, autocrine, paracrine, and endocrine pathways, with regulatory effects on recipient cells. EVs show a long circulation half-life, and they are well tolerated in the body, are internalized by cells, and are able to cross the blood–brain barrier. These peculiar features make them a good vehicle for drug delivery in specific target organs and tissues [36]. OMIC technologies are largely used to characterize EVs, deepening the knowledge about their roles and functions in pathological conditions and in host response to viral infections [48,49,50,51]. The typical content of EVs is reported in Figure 3.

Evidence from several studies highlights the involvement of EVs in the progression of viral infections and in the mechanism of viral evasion of the host immune system, such as those seen in some herpes viruses, the hepatitis C (HCV) virus, and the human immunodeficiency virus (HIV) [52,53,54]. To date, since the pandemic period, many scientific efforts have been directed toward the characterization of EVs’ role in the COVID-19 disease. In particular, SARS-CoV-2 cell-to-cell transmission could facilitate the virus’s escape from neutralizing antibodies [55]. 

Further investigations about the packaging of viral elements and the characterization of host factors with immunostimulatory properties inside EVs are needed, which will provide information that is particularly useful for developing new prophylactic approaches.

## 2. Involvement of EVs in Virus-Mediated Infections

In 2003, Gould et al. proposed “The Trojan exosome hypothesis”, which suggests that retroviruses could utilize exosomes to enter host cells, promote viral spread, and evade the immune response because exosomes and retroviral particles use the same protein targeting pathway and contain a similar array of host cell lipids and proteins, thereby playing a central role in the pathogenesis of diseases [56]. The role of EVs in viral infections is highly controversial. Several research studies identified a strategy to facilitate viral transmission in EVs by transporting viral biological materials as cargo in the cell–cell communication system. On the other hand, they also intervene in antiviral host response by stimulating the release of proinflammatory mediators in target cells and/or regulating viral replication, as shown for the human immunodeficiency virus (HIV) [57,58,59]. Thus, extracellular vesicles’ role in the spread of viral, parasitic, fungal, and bacterial infections has been reported, but further investigations are needed. Being carriers of biological information and fundamental mediators of cell–cell communication, EVs could promote or inhibit host immunity depending on their cargo. EVs can be released from infected cells, as well as from pathogens, and participate in the dissemination of microbial components. In fact, exosomes can interact with non-immune host cells that influence the severity of infection, such as endothelial cells and fibroblasts. Pathogens or their components (pathogen-associated molecular patterns, PAMPs) are capable of triggering the innate or acquired host response by activating the production of cytokines, chemokines, and other immune effector molecules, and by triggering antigen presentation. Obviously, over the course of their evolution, pathogens have evolved different strategies to evade the host immune system [60,61]. The involvement of EVs in HIV infection, as reported in the literature, highlights the ambiguity of their role. The chemokine receptors CCR5 (C-C chemokine receptor type 5) and CXCR4 (C-X-C motif chemokine receptor 4), which are critical in HIV infection, have been seen in the microparticles released from PBMCs (peripheral blood mononuclear cells) or megakaryocytes. Thus, releasing these receptors to uninfected cells make these cells more vulnerable to infection [62,63]. Instead, exosomes extracted from HIV-infected T cells contain trans-activation response element (TAR) RNA, which acts by downregulating apoptotic signals [64]. On the other hand, in contrast to immune inhibition, exosomes can exert a crucial role in viral replication inhibition. Indeed, host cell exosomes may contain the deaminase apolipoprotein B mRNA editing enzyme, catalytic subunit 3G (APOBEC3G), which is important for innate antiviral immunity and acts as an inhibitor of retrovirus replication and retrotransposon mobility via deaminase-dependent mechanisms, thus making viral replication much less efficient in recipient cells [65]. HIV transcriptional repression could be mediated by exosomes derived from infected CD8+ T lymphocyte cells [66]. A dual role of exosomes has also been demonstrated for Epstein–Barr virus (EBV). Latent membrane protein-1 (LMP-1) is present on the exosomes of infected cells, which can block T cell proliferation and inhibit the cytotoxic activity of natural killer (NK) cells, causing immunosuppressive effects, while Galectin-9, present in the exosomes released from EBV-infected cells, induces apoptosis of EBV-specific CD4+ T cells. On the other hand, several EBV miRNA are delivered by exosomes to induce the repression of host antiviral activity [67]. The implication of EVs in viral infections have been demonstrated for cytomegalovirus (CMW), hepatitis C virus (HCV), and herpes simplex virus (HSV).

It has been observed that the exosome-mediated LMP-1 release avoids its degradation and reduces NF-κB signaling, promoting the EBV persistence in B cells.

In addition to the aforementioned implications of EVs in viral infections, a point to better highlight is the virus’ ability to hijack the EV pathway to manage their cycle. Several works of scientific evidence have shown that components of the ESCRT system are implicated in both EV biogenesis and in virus life cycles processes, being the cellular compartments used for the exosomes biogenesis and the sites assigned to the viral assembly physically proximal. Some viruses enter the host cell through interactions with specific receptors, others by endocytosis, depending on their structure. At the end of their formation and assembly, viruses can leave the host cell either by exocytosis (HCV) or directly from the plasma membrane (HIV). HIV uses its Gag protein to recruit ESCRT-I and Alix components. Other literature evidence has reported that several members of herpes virus family (alpha HSV1, beta CMV and HSV6, gamma EBV, and Kaposi Sarcoma-associated herpesvirus) are able to remodel the exosome cargo coming from infected cells to their advantage. Concerning HSV1, in 2010 Temme et al. demonstrated the ability of HSV-1 encoded glycoprotein B (gB) to direct the HLA-DR (DR) molecules not on the cell surface but release into the exosomes pathway, affecting the viral immune evasion mechanisms. It has been proposed that HSV-1 promotes STING (stimulator of IFN genes) and viral mRNAs/microRNAs transport by exosomes, probably with the aim of silencing viral genes in latently infected neurons and to reduce the spread of infection. By the STING exosomes-mediated transport from infected to uninfected cells, the virus is able to manage their replication, facilitating person-to-person viral spread. On the other hand, miR-H28 and miR-H29 produced in the late phase of a productive viral infection, and significantly abundant in infected cells in latent reactivation phase, are transported within the exosomes. Their ectopic expression results in a slowdown of the HSV-1 infection, so the virus is able to modulate its expression and diffusion [68,69,70,71,72].

**Figure 4:**
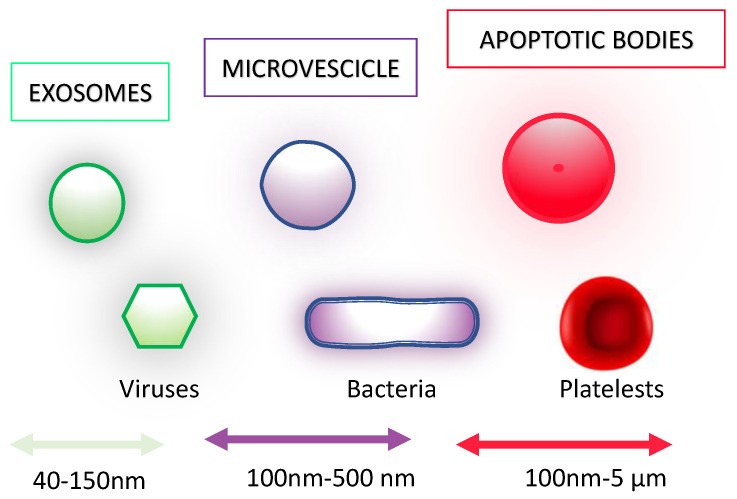
Extracellular vesicles’ size in comparison to viruses, bacteria, and platelets [75]. Viruses and exosomes not only share the same dimensions but also show the same characteristic of a lipid envelope (in the case of virus coated with spike proteins) and the capability to deliver genomic material and to ligate specific surface cell proteins.

Other data from the literature show that MΦ-EV (human vesicle-derived extracellular macrophages) can act as inhibitors of both viral replication and transmission. Exosomes produced by U937 macrophage cells infected with dengue virus 2 (DENV-2) are loaded with miRNA-regulating factors that are involved in host immune response (such as TNF-α) and viral protein (NS3), inducing a downstream protective response during the early stages of DENV infection [73]. Interferon resistance as a protective strategy, through the delivery of macrophage-released miRNA to hepatocytes, has also been observed for hepatitis B virus (HBV) [74] and hepatitis C virus (HCV) [75]. The sizes of EVs are reported in Figure 4.

## 3. EV-Mediated Regulation of Innate and Adaptive Immune Responses

EVs may contribute to both the activation of the adaptive immunological response and the suppression of inflammation. In particular, inflammatory suppression can occur by increasing Treg (lymphocyte T regulator) function, suppressing NK (natural killer) and CD8+ cell activities, and inhibiting monocyte differentiation into DCs (dendritic cells) and their subsequent maturation. Regarding the reported prominent role of EVs in the adaptive immune response, it is known that (i) EVs carry MHC class I [75] and class II [38] molecules [76,77,78]; (ii) EVs released by antigen-presenting cells (APCs) mediate the direct antigen presentation to T cells by the MHC class I and II receptors, stimulating CD4+ and CD8+ T cells [79]; and (iii) EVs present antigens in an indirect manner via APCs [77,78,79,80].

In the context of evidence of EVs as mediators of the immune response, previous research has highlighted their capability to interact and collaborate with cytokines. Similar to cytokines, EVs function as cellular messengers with both synergistic or antagonistic, pleiotropic, and redundant functions. A high level of serum cytokine production in COVID-19 patients is noted to be associated with the severity and progression of the disease. EVs can play the role of mediators in a receptor-dependent and/or -independent manner, both through the presence of cell surface receptors and as intracellular target molecules. Instead, cytokine function is limited and subjected to the presence of specific receptors expressed on the plasma membrane of target cells. EVs can deliver cytokines both internally and on their surface, with the advantage of cytokines being protected from enzymatic degradation in the extracellular environment and reaching distant target cells [81,82].

Dysregulation of the immune response with hyper-activation of the innate immune system, T-cell lymphopenia, and release of the cytokine storm, along with activation of neutrophils and macrophages as mediators of hyper-inflammation, are the presentation of severe cases of COVID-19. This severe clinical profile is also supported by an increase in serum inflammatory markers, such as erythrocyte sedimentation rate, C-reactive protein, ferritin, fibrinogen, D-dimer, and lactate dehydrogenase, together with higher levels of the cytokine interleukin-6 (IL-6) and other relevant biomarkers. In particular, clinical trials based on IL-6 signaling pathway intervention are underway for COVID-19 treatment [80,81,82,83].

EVs, such as those in viruses, are able to elicit immune reactions and/or attenuate inflammatory responses in the host. It is well known that EVs carry biological information of different natures and functions, including information on factors related to metabolism and inflammatory or stress response. A recent Italian study on exosomes derived from the plasma of COVID-19 patients, who were exposed to SARS-CoV-2 spike-derived fragments, has linked the burden of EVs to the severity of disease. In particular, Pesce et al. established that the levels of exosomes of MILD patients were more abundant than healthy donors (HD) and SEVERE patients, and that, independent from the analyzed exosomal markers, exosomes from COVID-19 patients had an increased size distribution compared to HD exosomes. The CD41a+ exosome subpopulation of MILD COVID-19 patients was larger than that of SEVERE patients. The inhomogeneous size could reflect the different cargos inside the exosomes. Furthermore, CD41a+ and CD9+ subpopulations from MILD patients were enriched with SARS-CoV-2-S peptides, whereas exosomes from MILD patients had SARS-CoV-2-S fragments with the spike protein’s RBD diffused on their surface. The MILD patients’ SARS-CoV-2-S+ exosomes were mostly of B cell, dendritic cell, and monocyte/macrophage origins, and were thus derived from APCs. This evidence highlights possible parentally conserved antigen-presenting functions; thus, exosomes could act as auto-sufficient antigen-presenting vehicles, with consequent modulation of antigen-specific T-cell responses. Compared to those derived from SEVERE COVID-19 patients, exosomes derived from MILD COVID-19 patients were more efficient in stimulating CD4+ T-cell activation and proliferation, with an induction of interleukin-2 (IL-2) secretion in vitro, which could trigger a rapid resolution of the disease in vivo. The ability of exosomes from MILD COVID-19 patients to act as strong stimulators of CD4+ T-cell activation and growth was confirmed by their proteome cargos, which were related to immune response, cell growth, signal transduction, and MHC class II receptor functionality; in contrast, exosomes derived from SEVERE COVID-19 patient correlated with immune/inflammatory responses (such as regulators of IL-6 proinflammatory signaling), members of the coagulation system, and protein metabolism functions. The exosome profiles of MILD and SEVERE COVID-19 patients were drastically different, showing different immunomodulatory functions. The EV cargo in MILD patients was mainly involved in immune cell activation, while for SEVERE patients, the EV cargo was principally involved in the inflammatory process [84]. In their recent work, El-Shennawy et al. (2022) [85] proposes a protective role of exosomes from SARS-CoV-2 infection. They observed a correlation between the increased level of EVs containing ACE2 (evACE2) in the plasma of positive COVID-19 patients and the disease severity. The data obtained also show that evACE2 are able to interact with the RBD protein more efficiently than the cellular ACE2. This competition results in a better neutralizing effect by evACE2 on SARS-CoV-2 and in a protective role in transgenic mice (hACE2) from lung damage and mortality, independently by the virus variant counteracted (α, β e δ). EvACE2’s role should be better investigated and characterized in order to find a strategy capable of implementing the prophylaxis in place and which can cover the subsequent variants of the virus [85].

**Figure 5:**
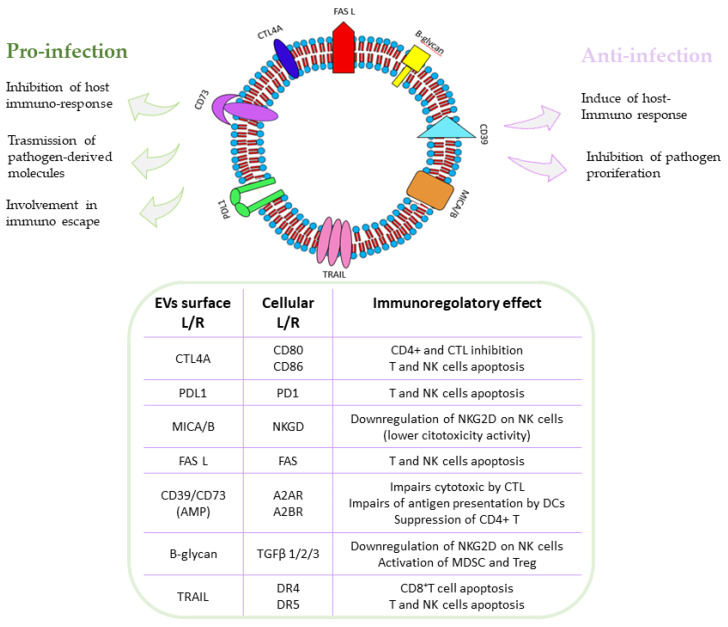
EVs’ proinflammatory and anti-inflammatory actions [63,64,65,66]. Abbreviations: PDL1: programmed cell death ligand 1; CTLA4: cytotoxic T lymphocyte antigen 4; FASL: apoptosis-inducing ligand; TRAIL: TNF-related apoptosis-inducing ligand; CD39: ectonucleoside triphosphate diphosphohydrolase-1; CD73: ecto-5′-nucleotidase; CTL: cytotoxic T lymphocyte; NK: natural killer; DCs: dendritic cells; TGFβ: immunosuppressive cytokine transforming growth factor-β; MDSCs: myeloid-derived suppressor cells; NKG2D: killer cell lectin-like receptor K1; MICA: MHC class I polypeptide-related sequence A; MICB: MHC class I polypeptide-related sequence B; L/R: ligand/receptor.

In addition to their proinflammatory role, an anti-inflammatory mechanism that promotes tissue repair and remodeling has been proposed for EVs, which is in line with their peculiar profile, as suggested by the presence of oxidized lipid derivatives in EVs and the changes in cellular lipid metabolism and inter-tissue crosstalk in different stages of COVID-19, underlying their potential use as a therapeutic treatment [86]. On the other hand, it is reported that thymic epithelial cell-derived EVs carry proteins involved in the maturation of single positive (CD4+ or CD8+) thymocytes [87]. B cell-derived EVs present MHCs on their surface, thus being able to directly present antigens to T cells [88]. The implication and the dual rule of EVs as immune mediators are shown in Figure 5.

## 4. Mesenchymal Stromal/Stem Cells (MSCs) and MSC-Derived Extracellular Vesicles

Mesenchymal stem cells, also known as mesenchymal stromal cells or medicinal signaling cells (MSCs), can be isolated from bone marrow (BM-MSCs), umbilical cord (UC-MSCs), adipose tissue (AD-MSCs), and many other biological sources, including dental tissue (dental pulp of deciduous teeth), amniotic fluid and membranes, the endometrium, skin and foreskin, and limb buds; each of these expresses a unique set of MSC markers, while CD90 represents a common MSC marker [89,90].

In 2019, the International Society for Cell & Gene Therapy (ISCT^®^) Mesenchymal Stromal Cell (ISCT MSC) committee offered a position statement to clarify the nomenclature of mesenchymal stromal cells (MSCs), in which the recommendation about the use of the acronym “MSCs” is specified [91]. Due to their ability to interact with immune cells via paracrine factor secretion with consequent immunomodulatory effects, their ease of isolation, and their large ex vivo expansion capacity, MSCs represent a good resource that can be better characterized in the therapeutic field. In fact, the therapeutic effects of MSCs in a wide spectrum of tissue/organ damage have already been investigated over the past few decades, in particular in inflammatory diseases. Above all, their potential therapeutic effects were investigated in lung diseases and other disorders, such as asthma [69,70], idiopathic pulmonary fibrosis (IPF) [91,92], ARDS [93,94], and chronic obstructive pulmonary disease (COPD) [95,96], such as in respiratory virus-induced lung infections [97,98]. Moreover, the evidence in the literature suggests an improvement in the survival rate of H7N9-induced ARDS through treatment with MSCs in both preclinical and clinical studies, similar to COVID-19-mediated ARDS; thus, MSCs present a good candidate for the development of a COVID-19 disease treatment strategy. [99] Already in 2018, a study by Bernard et al. investigated the protective action of MSCs in various mouse models of lung injury. Under distal lung damage conditions, local alveolar hypoxia promotes massive alveolar epithelial cell (AEC) apoptosis and AEC epithelial–mesenchymal transition. In cases of alteration in repair cellular mechanism due to damage, pulmonary diseases such as ARDS or idiopathic pulmonary fibrosis (IPF) can occur, which are typically associated with alveolar hypoxia. This research group verified that human MSCs exert a protective paracrine effect by limiting lung inflammation and fibrosis and preventing ROS accumulation and HIF-1α subunit stabilization, with subsequent downregulation of proapoptotic protein expression. This evidence proposes allogeneic mesenchymal stem cells (MSCs) as a promising therapeutic approach in pulmonary diseases such as IPF and ARDS [100]. Moreover, because of their immunomodulatory properties, MSCs represent a strategy to avoid or alleviate the cytokine storm, such as that reported in ARDS where MSCs play a role in clearing alveolar fluid disturbed by the cytokine storm in the lungs [101]. MSCs can affect both innate and adaptive immunity through the release of soluble factors, such as angiogenic factors, chemokines, cytokines, growth factors, and EVs, and/or through direct cell–cell contact [100,101]. It is noted that MSC-derived extracellular vesicles (MSC-EVs) are able to reduce the secretion of proinflammatory cytokines, thereby inhibiting the activation and proliferation of a variety of proinflammatory cells (Th1, Th17, and M1 macrophages) [102]. On the other hand, they promote the proliferation of anti-inflammatory cells (M2 macrophages and Tregs) [103,104], induce an increase in anti-inflammatory cytokine secretion, and can inhibit the function of natural killer cells [105] and the proliferation of peripheral blood mononuclear cells (PBMCs) [106,107]. Several studies reported that allogeneic UC-MSC administration is safe in a multitude of diseases, perhaps due to their low levels of class I and class II human leukocyte antigens, thus reducing alloreactivity [108,109,110]. In 2020, Lanzoni et al. proposed a double-blind, randomized study to establish safety and to explore the efficacy of allogeneic UC-MSC infusions in hospitalized patients with ARDS secondary to COVID-19; they reported that two intravenous infusions of UC-MSCs (dose of 100 million cells per infusion) were safe in COVID-19 patients with ARDS. Moreover, the effects of the treatment were demonstrated through both a significant decrease in a set of inflammatory cytokines and a significant improvement in patient survival and time to recovery. The evidence highlights the importance of UC-MSC administration, which requires further investigations to establish its efficacy and safety [111]. As of 4 August 2023, 52 clinical trials investigating the use of MSCs as a treatment for COVID-19 had been initiated or were completed, as reported on clinicaltrials.gov. The search strategy performed, using the key words of “COVID-19” and “MSC”, led to the discovery of 385 clinical trials. Nevertheless, only 52 trials used MSCs as a treatment, as identified from www.clinicaltrial.gov (accessed on 16 July 2023) and shown in Supplementary Table S1 [112].

### MSC-EVs in COVID-19

The identification and characterization of EVs as therapeutic approaches for several diseases is gaining traction, with their use as cargo delivery systems of specific miRNAs, mRNAs, and drugs to treat COVID-19. The evidence from several studies suggests MSC-EVs as the new frontiers in regenerative medicine [113,114]. In particular, due to their immunomodulatory and differentiation effects, MSC-EVs are implicated in the attenuation of autoimmune and inflammatory diseases, such as rheumatoid arthritis (RA), or in both pro-tumor and anti-tumor effects, such as in osteosarcoma progression; MSC-EVs also have pivotal roles as a cell-free therapy for liver diseases and acute kidney diseases or kidney injury (AKI), such as chronic kidney disease (CKD), diabetic nephropathy (DN), and atherosclerotic renovascular disease (ARVD) [115]. Through the regulation of VEGF (vascular endothelial growth factor), HGF (hepatocyte growth factor), FGF7 (fibroblast growth factor 7), and the TGF-signaling pathways, MSC-EVs are involved in the repair of damaged tissues. Thus, they are good candidates to block or relieve COVID-19 patients’ symptoms. Recent work by Nawaz M. et al. (April 2023) proposed the delivery of VEGF-A mRNA by lipid nanoparticles (LNPs). Surprisingly, they showed that translation of the delivered exogenous mRNA begins immediately and that a fraction of internalized VEGF-A mRNA is secreted via EVs. Furthermore, several proangiogenic transcripts are overexpressed in EVs obtained from cells overexpressing VEGF-A mRNA. This study shows the ability of LNPs to transform EVs as functional extensions to distribute therapeutic mRNA among cells in a manner different from LNPs [116].

In another study, allogeneic exosomes derived from bone marrow mesenchymal stem cells (ExoFlo™) were investigated as a treatment for severe COVID-19. A single dose of ExoFlo (15 mL via the IV route) was infused into 24 critical patients with COVID-19. The experimentation showed an improvement in several biochemical parameters and in the immune system response. In particular, the MSC exosome infusion was able to reduce reactive C-protein (CRP), D-dimer, and ferritin levels, leading to a stabilization of neutrophil/lymphocyte count, downregulation of the cytokine storm, and restoration of oxygenation in patients with clinical severe conditions, with no adverse effects [117].

**Figure 6:**
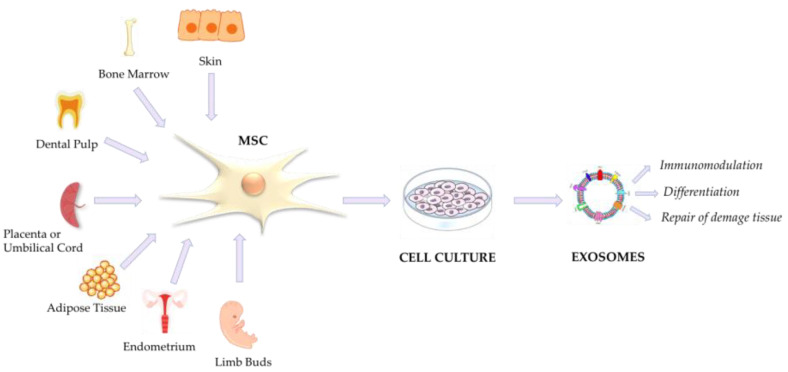
Mesenchymal stem cell-derived exosomes from different tissues and their implication in COVID-19 treatment [115].

A recent pilot trial (Meiping Chu et al., 2022) investigated a nebulization therapy using UC-MSC-derived exosomes for COVID-19 pneumonia. The interesting results demonstrated that the nebulization approach was simple, safe, and did not induce acute allergic or secondary allergic reactions. Moreover, the therapy was effective in reducing time of hospitalization and promoting the absorption of pulmonary lesions in mild cases of COVID-19 pneumonia (Chinese Clinical Trial Registry, ChiCTR2000030261. Registered on 26 February 2020) [118]. Another recent Chinese study tested the use of aerosol inhalation of exosomes derived from human adipose-derived MSCs (haMSC-Exos) on seven patients with severe COVID-19-related pneumonia (four males and three females) for 5 consecutive days. The haMSC-Exos inhalation was well tolerated, with no evidence of prespecified adverse events, which was accompanied by an improvement in CT imaging within 7 days [119]. A schematic representation of MSC exosomes derived from different biological matrixes and their implication in COVID-19 is shown in Figure 6.

As of 4th August 2023, clinical trials involving exosomes as a therapeutic approach for the treatment of COVID-19 were in progress, of which four trials were based on MSC-derived exosomes, as reported in Table 2 [120] (http://www.clinicaltrials.gov) (accessed on 16 July 2023). Two clinical trials (NCT04276987 and NCT04313647) investigating the use of the inhalation of exosomes derived from allogeneic adipose mesenchymal stem cells for the treatment of COVID-19 pneumonia, as well as (NCT04313647) evaluating its safety and tolerance in healthy-looking volunteers, had already concluded.

Exosomes show the advantages of being able to bypass biological barriers, acting as drug delivery systems, and being engineerable with the insert of various biological information and/or drugs, such as remdesivir used for COVID-19 treatment; exosomes can be obtained from patients’ plasma, which can be helpful in preventing reject phenomena [121]. Another useful approach is to deliver regulatory mRNA against the SARS-CoV-2 gene via exosomes to switch off fundamental viral expressions [122]. Evidence in the literature has shown that an excess of soluble ACE2 can bind the spike S protein on SARS-CoV-2 virus, blocking its fusion to the host membrane, with a viral neutralizing effect. This competition for S can be exploited as a competitive inhibition therapy to engineer MSC-derived exosomes, leading to the overexpression of ACE2, thereby sequestering viral components. The small number of patient populations analyzed in the various studies represents a major limitation in the characterization of MSC-EV applications. Also, the route, source, and period window of administration, the optimal dose and dose frequency, and side effects should be well evaluated, confirmed, and established.

## 5. Translational EV Application in Diagnostical/Therapeutic Approaches

Exosomes can be recovered from several biological matrixes, such as saliva, blood, urine, tears, milk, and other sources. The exosomal component varies in qualitative and quantitative terms between healthy individuals and those with underlying diseases, including tumors, neurological disorders, and infectious diseases [123]. For example, Welker et al. correlated the abundance of the exosomal protein CD81 with higher levels of alanine transaminase (ALT, a marker of liver damage) and more severe liver fibrosis, showing that CD81 measurement is useful in diagnosing or monitoring the course of chronic hepatitis C infection [92]. Balbi et al. [124], showed for the first time the antigenic profiling of circulating EVs in COVID-19 patients. The specific EVs surface marker characterization (CD49e, CD209, CD69, CD142, and CD20) allowed for the drawing of a defined EV signature, useful to discriminate between patients negative for SARS-CoV-2 who experienced infection symptoms and those with positive clinical status of the infection. The signature also resulted in differences between patients with pneumonia COVID-19 negative versus those with pneumonia but positive on the SARS-CoV-2 nasopharyngeal swab test. CD142 (platelet tissue factor (TF)), implicated in the extrinsic pathway of the blood coagulation cascade, was found to be the most expressed EVs marker in SARS-CoV-2-positive patients and its expression levels increase directly correlates with the duration of hospitalization. Interestingly, CD142 on EVs’ surface showed biological activity, and most of the vesicles obtained resulted from endothelial or platelet origin, leading to the evaluation of a strong implication of EVs in coagulation disorders in COVID-19 patients, maybe contributing to the disease severity. The authors promote the signature identified as a powerful tool of potential prognostic biomarkers. On the other hand, the prothrombotic activities of EVs have been greatly investigated, as reported in reviews about the specific topic [124,125].

The Cappellano et al. study (2021) [126] suggests the evaluation of the circulating platelets (PLT)-derived extracellular vesicles (EVs) pattern as a rapid identification test of SARS-CoV-2 infection, starting from unmanipulated blood samples. They found that the pattern of circulating PLT-EVs is altered in COVID patients. In particular, PLT-EVs counts were higher in SARS-CoV-2-positive patients compared to negative controls, aside from being highly associated with SARS-CoV-2 infection [126].

These results, together with other scientific evidence, promote the characterization and subsequent use of exosomal components as diagnostic and prognostic disease biomarkers to trace the differences between healthy individuals and affected patients.

The intrinsic characteristics of exosomes make them excellent candidates as vehicles for vaccine production. Firstly, after being released in body fluids, they can reach different body sites that are very far from each other, in addition to their ability to cross the blood–brain barrier, and they could be engineered to deliver in a pre-established manner. In the oncological field, autologous exosomes have been investigated for their possible applications as tumor cell-based exosome vaccines, such as exosome-based immunotherapy, promising an alternative nanomedicine immunotherapeutic approach [127,128]. Other than the above-cited MSC-derived exosome therapeutic approaches, the cutting-edge approaches of exosome-based vaccines as new frontiers to develop anti-SARS-CoV-2 vaccines are supported by evidence that correlates the presence and levels of exosomes after mRNA vaccination. According to the data produced by Bansal S. et al., the exosomes with exposed viral S antigenic protein circulated following mRNA vaccination, along with the appearance of antibodies and T cells secreting IFN-γ and TNF-α, increased after a booster dose [129]. Another interesting approach using intranasal immunization of outer membrane vesicles derived from Salmonella typhimurium and decorated with mammalian cell culture-derived RBD (RBD-OMVs) showed high titers of blood anti-RBD IgG neutralizing antibody both against wildtype and Delta variants in animal models [130].

Whang Z. et al. proposed lung spheroid cell (LSC)-derived exosomes (LSC-Exo) conjugated to the recombinant SARS-CoV-2 receptor-binding domain (RBD) as a virus-like particle (VLP)-based lyophilized vaccine. This approach, which was tested on animal models, presents the advantage of being inhalable, and thus only non-invasive administration is required; it enhances the retention of the RBD in both mucus-lined and lung parenchyma cells in comparison to liposomes, and it is stable at room temperature for over three months after lyophilization. Moreover, by triggering an important mucosal T-cell response, this approach confers protection to the upper respiratory tract, which is important for controlling infection spread. However, it has limitations in terms of the robust production of exosomes and the variation in cargos and surface ligands of exosomes in the different batches produced. In particular, EVs can cargo SARS-CoV-2 antigens or mRNA to induce an immune host response through the activation of CD8(+) T cells and B cells in a targeted and specific way. The immune response is activated based on a vaccine strategy without the use of the virus [131]. In the same vein as the work cited above, Popowski K.D. et al. proposed an inhalable dry powder based on mRNA-loaded lung-Exos (S-Exos) coding for SARS-CoV-2 S protein that is able to elicit immunoglobulin G (IgG) and secretory IgA (SIgA) responses [132]. A multivalent protein-based vaccine consisting of exosomes designed to express the spike of the SARS-CoV-2 delta virus (Stealth X-Spike [STX-S]) or nucleocapsid protein (Stealth X-Nucleocapsid [STX-N]) on the surface generates potent humoral and cellular immune responses in mice and rabbits, with the advantage of administration of only nanograms of protein and without the use of an adjuvant [133]. The 2018 the “Minimal information for studies of extracellular vesicles” (MISEV) guidelines collected and reported evidence on extracellular vesicle nomenclature, ways to collect and pre-process EVs, their separation and concentration characterizations, functional studies, and other general considerations [28]. In 2021, the International Society for Extracellular Vesicles published a position statement, updating the MISEV2018 guidelines [134]. Unfortunately, this update reported many still unsolved critical issues and the need to perform more studies to optimize the guidelines for the management of EVs and invited the scientific community to collect specific information.

One fact for sure is that the literature is becoming increasingly rich in evidence relating the world of EVs to many diseases and promoting their versatile use and greater characterization.

## 6. Conclusions

The discovery, study, and characterization of EVs have opened a new chapter of translational and precision medicine through the exchange of information and the development of new approaches alternative to canonical ones (nanomedicine). The management of EVs has several limitations that still need to be understood and completely explored. The yield and standard isolation method of cell-derived EVs are in continuous evolution and remain difficult. The quantification of EVs can be influenced by the cell culture conditioned media, the starting biological matrix (cells number and/or size), and the samples storage conditions, parameters that should be indicated for each experimental use. However, the use of cutting-edge technologies is illuminating all aspects of this micro-world, which, for a long time, has been hampered by the lack of cutting-edge technologies capable of separating and characterizing EVs. Because of their ability to modulate the immune host response and their anti-inflammatory activity, EVs are a basis for the development of new biomarkers or therapeutic platforms. Since EVs carry biological information and materials that are derived from pathogens, they are promising diagnostic markers of infectious diseases, a field that has not yet been sufficiently investigated. Managing the cell-to-cell exosome-mediated communication could be a good therapeutic strategy. All efforts of the scientific community must continue to channel the knowledge obtained into the creation and marketing of vaccines that are safe, reliable, and stable, without requiring particular methods of storage or handling. Above all, they must be able to produce an efficient and long-lasting immune response, without the need for numerous booster shots, and be able to cover not only the already discovered variants but also represent a support for the rapid development of vaccines against potential novel variants. The nano- and sub-micrometric universe could represent a chance to better characterize future enemies and to achieve the best-performing vaccination strategies, and the evaluation of the secretome represents our hidden weapon.

## Figures and Tables

**Table 1:** Schematic representation of extracellular vesicle features.

	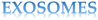	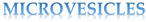	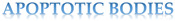
**Dimension (nm)**	40–150	40–10,000	100–5000
**Density (g/mL)**	1.13–1.19	Unknown	1.16–1.28
**Markers**	CD9, CD63, CD81, CD106, ICAM, Tspan8, Tspan29, Tspan30, TSG101, MFGE8	Integrins, selectins, CD82, CD40L, fibronectin, annexin, flotillin-2	Phosphatidylserine, annexin V
**Biogenesis**	via inward budding of the endosomal membrane	via outward budding of the cellular membrane	via membrane eversion of an apoptotic cell
**Cargo**	RNA, miRNA, ALIS, Peroxidases, G proteins, clathrin, VPS32, VPS4, HSP70, HSP90	RNA, miRNA, Tau, TDP43, GAPDH, ARF6, Erk, PLD, HSP70, HSP90, actin, tubulin	DNA, RNA, miRNA, other ncRNAs, histones, cytoplasmatic proteins, organelles
**Functional properties**	Selective cargo transfer, receptor interaction, immune response	Coagulation, thrombosis, angiogenesis, tissue regeneration, inflammation	Transfer of DNA fragments to phagocytes, cell survival, inhibition of inflammatory process
**TEM morphology**	Cup-shaped	Cup-shaped	Heterogeneous

**Table 2:** Clinical trials investigating the use of MSC-derived exosomes in COVID-19 patients (www.clinicaltrials.gov) (accessed on 16 July 2023). Abbreviations: UK: Unknown; NYR: Not_Yet_Recruiting; C: Completed; R: Recruiting; I: Interventational.

NCT Number	Study Title	Study Status	Study Type	Interventions
**NCT04798716**	The Use of Exosomes for the Treatment of Acute Respiratory Distress Syndrome or Novel Coronavirus Pneumonia Caused by COVID-19	NYR	I	DRUG: MSC-derived exosomes delivered intravenously every other day using an escalating dose (2:4:8)|DRUG: MSC-derived exosomes delivered intravenously every other day using an escalating dose (8:4:8)|DRUG: MSC-derived exosomes delivered intravenously every other day (8:8:8)
**NCT05808400**	Safety and Efficacy of Umbilical Cord Mesenchymal Stem Cell Exosomes in Treating Chronic Cough After COVID-19	R	I	BIOLOGICAL: MSC-derived exosomes
**NCT04602442**	Safety and Efficiency of Method of Exosome Inhalation in COVID-19 Associated Pneumonia	UK	I	DRUG: EXO 1 inhalation|DRUG: EXO 2 inhalation|DRUG: Placebo inhalation
**NCT05787288**	A Clinical Study on Safety and Effectiveness of Mesenchymal Stem Cell Exosomes for the Treatment of COVID-19.	R	I	BIOLOGICAL: Extracellular vesicles from mesenchymal stem cells
**NCT05216562**	Efficacy and Safety of EXOSOME-MSC Therapy to Reduce Hyper-inflammation In Moderate COVID-19 Patients	R	I	DRUG: MSC-derived exosome intravenous injection|DRUG: Placebo intravenous injection|DRUG: COVID-19 standard treatment
**NCT04491240**	Evaluation of Safety and Efficiency of Method of Exosome Inhalation in SARS-CoV-2 Associated Pneumonia.	C	I	DRUG: EXO 1 inhalation|DRUG: EXO 2 inhalation|DRUG: Placebo inhalation
**NCT04747574**	Evaluation of the Safety of CD24-Exosomes in Patients With COVID-19 Infection	UK	I	DRUG: EXO-CD24

## Data Availability

Data are contained within the article.

## Supplementary Materials

The following supporting information can be downloaded at: https://www.mdpi.com/article/10.3390/v15101976/s1, Table S1: Clinical trials about the involvement of MSC in COVID-19 disease from www.clinicaltrials.gov. Abbreviations: WD: Withdrawn; UK: Unknown; AV: Available; T: Terminated; NYR: Not_Yet_Recruiting; C: Completed; ANR: Active_Not_Recruiting; R: Recruiting; NLA: No_Longer_Available; I: Interventational; E_A: Expanded_Access; O: Observational.
