# Supplementary material for: Extracellular Vesicles as a Translational Approach for the Treatment of COVID-19 Disease: An Updated Overview
Source: Viruses. 2023 Sep 22;15(10):1976. doi: 10.3390/v15101976 (PMC10611252; doi:10.3390/v15101976)
Supplement: Supplementary file 1 [file viruses-15-01976-s001.zip › viruses-2580671-supplementary.pdf]

Table S1. Clinical trials about the involvement of MSC in COVID-19 disease from www.clinicaltrials.gov. Abbreviations: WD: WITHDRAWN; UK: UNKNOWN; AV: AVAILABLE; T: TERMINATED; NYR: NOT_YET_RECRUITING; C: COMPLETED; ANR: ACTIVE_NOT_RECRUITING; R: RECRUITING; NLA: NO_LONGER_AVAILABLE; I: INTERVENTATIONAL; E_A: EXPANDED_ACCESS; O: OBSERVATIONAL.

NCT Number	Study Title	Study Status	Interventions	Study Type
**NCT04909879**	Study of Allogeneic Adipose-Derived Mesenchymal Stem Cells for Non-COVID-19 Acute Respiratory Distress Syndrome	WD	BIOLOGICAL: COVI-MSC|DRUG: Placebo	I
**NCT04429763**	Safety and Efficacy of Mesenchymal Stem Cells in the Management of Severe COVID-19 Pneumonia	UK	BIOLOGICAL: Umbilical cord derived mesenchymal stem cells|BIOLOGICAL: Placebo	I
**NCT04444271**	Mesenchymal Stem Cell Infusion for COVID-19 Infection	UK	DRUG: Mesenchymal stem cells|OTHER: Placebo	I
**NCT04416139**	Mesenchymal Stem Cell for Acute Respiratory Distress Syndrome Due for COVID-19	UK	BIOLOGICAL: Infusion IV of Mesenchymal Stem cells	I
**NCT04456439**	Intermediate-size Expanded Access Program (EAP), Mesenchymal Stromal Cells (MSC) for Multisystem Inflammatory Syndrome in Children (MIS-C) Associated With Coronavirus Disease (COVID-19)	AV	BIOLOGICAL: Remestemcel-L|DRUG: Hydrocortisone|DRUG: Diphenhydramine	E_A
**NCT04903327**	Study of Intravenous COVI-MSC for Treatment of COVID-19-Induced Acute Respiratory Distress	T	BIOLOGICAL: COVI-MSC|DRUG: Placebo	I
**NCT04371393**	MSCs in COVID-19 ARDS	T	BIOLOGICAL: Remestemcel-L|DRUG: Placebo	I
**NCT04869397**	Treatment of Respiratory Complications Associated With COVID-19 Using Umbilical Cord Mesenchymal Stromal Cells	T	BIOLOGICAL: Allogeneic Wharton's jelly-MSCs (WJ-MSC)|OTHER: Placebo	I
**NCT04452097**	Use of hUC-MSC Product (BX-U001) for the Treatment of COVID-19 With ARDS	NYR	BIOLOGICAL: Human umbilical cord mesenchymal stem cells + best supportive care|OTHER: Placebo control + best supportive care	I
**NCT04299152**	Stem Cell Educator Therapy Treat the Viral Inflammation in COVID-19	UK	COMBINATION_PRODUCT: Stem Cell Educator-Treated Mononuclear Cells Apheresis	I
**NCT04390152**	Safety and Efficacy of Intravenous Wharton's Jelly Derived Mesenchymal Stem Cells in Acute Respiratory Distress Syndrome Due to COVID 19	UK	DRUG: Wharton's jelly derived Mesenchymal stem cells.|DRUG: Hydroxychloroquine, lopinavir/ritonavir or azithromycin and placebo (standard therapy)	I
**NCT04445220**	A Study of Cell Therapy in COVID-19 Subjects With Acute Kidney Injury Who Are Receiving Renal Replacement Therapy	UK	BIOLOGICAL: SBI-101	I
**NCT04445454**	Mesenchymal Stromal Cell Therapy for Severe Covid-19 Infection	R	BIOLOGICAL: Mesenchymal stromal cells	I
**NCT04865107**	Cellular Immuno-Therapy for COVID-19 ARDS Randomized Clinical Trial	UK	BIOLOGICAL: UC-MSCs|BIOLOGICAL: Placebo	I
**NCT04992247**	Study of Allogeneic Adipose-Derived Mesenchymal Stem Cells to Treat Post COVID-19 "Long Haul" Pulmonary Compromise	NYR	BIOLOGICAL: COVI-MSC|BIOLOGICAL: Placebo	I
**NCT04382547**	Treatment of Covid-19 Associated Pneumonia With Allogenic Pooled Olfactory Mucosa-derived Mesenchymal Stem Cells	C	BIOLOGICAL: Allogenic pooled olfactory mucosa-derived mesenchymal stem cells|OTHER: Standard treatment according to the Clinical protocols	I
**NCT04333368**	Cell Therapy Using Umbilical Cord-derived Mesenchymal Stromal Cells in SARS-CoV-2-related ARDS	C	BIOLOGICAL: Umbilical cord Wharton's jelly-derived human|OTHER: NaCl 0.9%	I
**NCT04535856**	Therapeutic Study to Evaluate the Safety and Efficacy of DW-MSC in COVID-19 Patients	C	DRUG: allogeneic mesenchymal stem cell|OTHER: Placebo	I
**NCT04611256**	Mesenchymal Stem Cells in Patients Diagnosed With COVID-19	UK	BIOLOGICAL: MSC|DRUG: Control	I

**NCT04467047**	Safety and Feasibility of Allogenic MSC in the Treatment of COVID-19	UK	BIOLOGICAL: Mesenchymal Stromal Cells infusion	I
**NCT04494386**	Umbilical Cord Lining Stem Cells (ULSC) in Patients With COVID-19 ARDS	ANR	BIOLOGICAL: Umbilical Cord Lining Stem Cells (ULSC)丨OTHER: Placebo (carrier control)	I
**NCT05286255**	Mesenchymal Stromal Cells for COVID-19 and Viral Pneumonias	NYR	BIOLOGICAL: Allogeneic Mesenchymal Stromal Cells	I
**NCT04720612**	COVID-19 Immunologic Antiviral Therapy With Omalizumab	ANR	BIOLOGICAL: Omalizumab丨OTHER: Placebo	I
**NCT04366830**	Intermediate-size Expanded Access Program (EAP), Mesenchymal Stromal Cells (MSC) for Acute Respiratory Distress Syndrome (ARDS) Due to COVID-19 Infection	NLA	DRUG: Remestemcel-L	E_A
**NCT05240430**	When to Apply to Which Patient in MSC?	R	DRUG: Mesenchymal Stem Cell Antigen-1, Human	O
**NCT04565665**	Cord Blood-Derived Mesenchymal Stem Cells for the Treatment of COVID-19 Related Acute Respiratory Distress Syndrome	R	OTHER: Best Practice丨BIOLOGICAL: Mesenchymal Stem Cell	I
**NCT05787288**	A Clinical Study on Safety and Effectiveness of Mesenchymal Stem Cell Exosomes for the Treatment of COVID-19.	R	BIOLOGICAL: Extracellular Vesicles from Mesenchymal Stem Cells	I
**NCT05132972**	Allogenic UCMSCs as Adjuvant Therapy for Severe COVID-19 Patients	R	BIOLOGICAL: Normoxic Allogenic UCMSC丨OTHER: Normal saline solution	I
**NCT05348772**	A First-In-Human Phase 1b Study of AmnioPul-02 in COVID-19 / Other LRTI	C	DRUG: AmnioPul-02	I
**NCT04625738**	Efficacy of Infusions of MSC From Wharton Jelly in the SARS-Cov-2 (COVID-19) Related Acute Respiratory Distress Syndrome	C	BIOLOGICAL: Ex vivo expanded Wharton's Jelly Mesenchymal Stem Cells丨BIOLOGICAL: Placebo	I
**NCT04456361**	Use of Mesenchymal Stem Cells in Acute Respiratory Distress Syndrome Caused by COVID-19	ANR	BIOLOGICAL: Mesenchymal Stem Cells derived from Wharton Jelly of Umbilical cords	I
**NCT04898088**	A Proof of Concept Study for the DNA Repair Driven by the Mesenchymal Stem Cells in Critical COVID-19 Patients	C	BIOLOGICAL: Mesenchymal Stem Cells Transplantation	I
**NCT04399889**	hCT-MSCs for COVID19 ARDS	T	BIOLOGICAL: Human cord tissue mesenchymal stromal cells (hCT-MSC) manufactured by Duke University.丨OTHER: Placebo	I
**NCT04896853**	Treatment of Respiratory Complications Associated With COVID19,Influenza ,Metapneumovirus,RSV Infection Using ProTransÂ®	R	BIOLOGICAL: ProTransÂ®	I
**NCT04615429**	Clinical Trial to Assess the Efficacy of MSC in Patients With ARDS Due to COVID-19	R	BIOLOGICAL: Mesenchymal stromal cells丨OTHER: Placebo	I
**NCT04400032**	Cellular Immuno-Therapy for COVID-19 Acute Respiratory Distress Syndrome	C	BIOLOGICAL: Mesenchymal Stromal Cells	I
**NCT04355728**	Use of UC-MSCs for COVID-19 Patients	C	BIOLOGICAL: Umbilical Cord Mesenchymal Stem Cells + Heparin along with best supportive care.丨OTHER: Vehicle + Heparin along with best supportive care	I
**NCT04392778**	Clinical Use of Stem Cells for the Treatment of Covid-19	C	BIOLOGICAL: MSC Treatment丨BIOLOGICAL: Saline Control	I
**NCT04525378**	MSC-based Therapy in COVID-19-associated Acute Respiratory Distress Syndrome	UK	OTHER: Mesenchymal stromal cell-based therapy	I
**NCT04345601**	Mesenchymal Stromal Cells for the Treatment of SARS-CoV-2 Induced Acute Respiratory Failure (COVID-19 Disease)	C	BIOLOGICAL: Mesenchymal Stromal Cells丨OTHER: Supportive Care	I
**NCT04905836**	Study of Allogeneic Adipose-Derived Mesenchymal Stem Cells for Treatment of COVID-19 Acute Respiratory Distress	R	BIOLOGICAL: COVI-MSC丨DRUG: Placebo	I
**NCT04397796**	Study of the Safety of Therapeutic Tx With Immunomodulatory MSC in Adults With COVID-19 Infection Requiring Mechanical Ventilation	ANR	BIOLOGICAL: BM-Allo.MSC丨BIOLOGICAL: Placebo	I

**NCT04728698**	Study of Intravenous Administration of Allogeneic Adipose-Derived Mesenchymal Stem Cells for COVID-19-Induced Acute Respiratory Distress	WD	DRUG: COVI-MSC|DRUG: Placebo	I
**NCT05465798**	Beta-glucans for Hospitalised Patients With COVID-19	NYR	DRUG: MC 3x3|DRUG: Placebo	I
**NCT05741099**	Application and Research of Mesenchymal Stem Cells in Alleviating Severe Development of COVID-19 Infection	R	BIOLOGICAL: Umbilical cord mesenchymal stem cells implantation|OTHER: Comparator	I
**NCT04573270**	Mesenchymal Stem Cells for the Treatment of COVID-19	C	BIOLOGICAL: PrimePro|OTHER: Placebo	I
**NCT04909892**	Study of Allogeneic Adipose-Derived Mesenchymal Stem Cells to Treat Post COVID-19 "Long Haul" Pulmonary Compromise	WD	BIOLOGICAL: COVI-MSC	I
**NCT04482699**	RAPA-501-Allo Therapy of COVID-19-ARDS	T	BIOLOGICAL: RAPA-501-Allo off-the-shelf Therapy of COVID-19|OTHER: Placebo	I
**NCT04753476**	Treatment of Severe COVID-19 Patients Using Secretome of Hypoxia-Mesenchymal Stem Cells in Indonesia	UK	BIOLOGICAL: Injection of Secretome-MSCs|DRUG: Standard treatment of Covid-19	I
**NCT04466098**	Multiple Dosing of Mesenchymal Stromal Cells in Patients With ARDS (COVID-19)	ANR	BIOLOGICAL: Mesenchymal stromal cells|OTHER: Placebo	I
**NCT05433298**	Mesenchymal Stromal Cells for the Treatment of Patients With COVID-19.	WD	BIOLOGICAL: Mesenchymal stem cell|OTHER: Placebo	I
**NCT04377334**	Mesenchymal Stem Cells (MSCs) in Inflammation-Resolution Programs of Coronavirus Disease 2019 (COVID-19) Induced Acute Respiratory Distress Syndrome (ARDS)	NYR	BIOLOGICAL: MSC	I
